# Supplementary material for: Analysis of PPARγ Signaling Activity in Psoriasis
Source: Int J Mol Sci. 2021 Aug 10;22(16):8603. doi: 10.3390/ijms22168603 (PMC8395241; doi:10.3390/ijms22168603)
Supplement: Supplementary file 1 [file ijms-22-08603-s001.zip › Supplemental materials_Analysis of PPARg signaling activity in psoriasis/Pathway models/Models images and html files/Model of PPARG signaling in psoriasis/1012568249.html]

CASP1 --+> IL18


# ProtModification CASP1 --+> IL18

|  |  |
| --- | --- |
| URN | urn:agi-protmodification:in-out:urn:agi-llid:834:out:urn:agi-llid:3606:eff=positive:mch=cleavage |
| Connectivity | 2 |
| References | 274 |
| Effect | positive |
| Mechanism | cleavage |
| ObjectType | ProtModification |
| RelationArity | 2 |
| RelationNumberOfReferences | 170 |
| RelationSymbolicName | CASP1}OID --+> IL18}OID |
| RelationConfidenceLevel | 3 |
| RelationParticipantTypes | Protein -> Protein |
| Original # of References | 91 |

---
